# Maternal inflammation activated ROS-p38 MAPK predisposes offspring to heart damages caused by isoproterenol via augmenting ROS generation

**DOI:** 10.1038/srep30146

**Published:** 2016-07-22

**Authors:** Qi Zhang, Yafei Deng, Wenjing Lai, Xiao Guan, Xiongshan Sun, Qi Han, Fangjie Wang, Xiaodong Pan, Yan Ji, Hongqin Luo, Pei Huang, Yuan Tang, Liangqi Gu, Guorong Dan, Jianhua Yu, Michael Namaka, Jianxiang Zhang, Youcai Deng, Xiaohui Li

**Affiliations:** 1 Institute of Materia Medica, College of Pharmacy, Third Military Medical University, Chongqing 400038, China; 2 Center of Translational Medicine, College of Pharmacy, Third Military Medical University, Chongqing 400038, China; 3 The Center for Disease Control and Prevention of Chengdu Military Command, Chengdu 610021, China; 4 Division of Hematology, Department of Internal Medicine, The Ohio State University, Columbus, Ohio 43210, USA; 5 Colleges of Pharmacy and Medicine, University of Manitoba, Apotex Center 750, McDermot Avenue, Winnipeg, R3E 0T5, MB, Canada; 6 Joint Laboratory of Biological Psychiatry between Shantou University Medical College and the College of Medicine University of Manitoba, Shantou 515063, China

## Abstract

Maternal inflammation contributes to the increased incidence of adult cardiovascular disease. The current study investigated the susceptibility of cardiac damage responding to isoproterenol (ISO) in adult offspring that underwent maternal inflammation (modeled by pregnant Sprague-Dawley rats with lipopolysaccharides (LPS) challenge). We found that 2 weeks of ISO treatment in adult offspring of LPS-treated mothers led to augmented heart damage, characterized by left-ventricular systolic dysfunction, cardiac hypertrophy and myocardial fibrosis. Mechanistically, prenatal exposure to LPS led to up-regulated expression of nicotinamide adenine dinucleotide phosphate (NADPH) oxidases, antioxidant enzymes, and p38 MAPK activity in left ventricular of adult offspring at resting state. ISO treatment exaggerated ROS generation, p38 MAPK activation but down-regulated reactive oxygen species (ROS) elimination capacity in the left ventricular of offspring from LPS-treated mothers, while antioxidant N-acetyl-L-cysteine (NAC) reversed these changes together with improved cardiac functions. The p38 inhibitor SB202190 alleviated the heart damage only via inhibiting the expression of NADPH oxidases. Collectively, our data demonstrated that prenatal inflammation programs pre-existed ROS activation in the heart tissue, which switches on the early process of oxidative damages on heart rapidly through a ROS-p38 MAPK-NADPH oxidase-ROS positive feedback loop in response to a myocardial hypertrophic challenge in adulthood.

Cardiovascular disease (CVD) has become the leading global cause of death, accounting for 31% of deaths worldwide. Approximately 17.5 million patients die from CVD every year with the number being expected to increase to over 23.6 million by 2030^1^. Furthermore, the costs associated with the treatment and prevention of CVD are recognized as one of the most costly diseases to the health care system in relation to all the chronic non-infectious diseases^2^. Although treatment strategies based on traditional risk factors, such as lipid lowering and blood pressure control, have great beneficial effects in secondary prevention of CVD, the incidence and mortality of CVD continue to rise rapidly, worldwide^1^. This indicates that other subtle factors such as exposure to an inflammatory stimulus during pregnancy may also contribute to the degree and severity of CVD experienced in adulthood.

In the modern high-paced lifestyle, psychosocial stress has been proven to be closely connected to the increase of CVD incidence^3^. As one major component of auto-regulatory response to stress, activation of sympathetic nervous system (SNS) is the key trigger of stress-induced cardiovascular disease^4^,^5^. For example, post-ganglionic muscle sympathetic nerve activity has been reported to contribute to stress-induced cardiomyopathy in post-menopausal women following severe emotional stress^6^. Therefore, activation of SNS could be considered as a potential second hit when combined with other risk factors such as prenatal detrimental exposure contributes to the development of heart disease. Positive inotropic action of SNS activation in myocardium is mainly mediated by β-adrenergic receptors (β-ARs) in heart, which could also be activated by sympathomimetic amine agent isoproterenol (ISO)^7^. Persistent activation of SNS in the heart by ISO could evoke cardiac hypertrophy, myocardial fibrosis and cardiac function injury. As such, this represents a commonly well-accepted method that has been utilized in animals to induce various cardiac diseases^7^.

Developmental re-programming resulting from early life exposure to adverse stimuli is increasingly recognized as an important risk origin for adult CVDs^8^,^9^,^10^. Inflammatory challenge during pregnancy, such as bacterial infection^11^ and influenza^12^, has been proven to contribute to the increased incidence of adult CVD. We previously found that prenatal exposure to inflammatory stimulus led to offspring’s hypertension at an elder age by challenging pregnant Sprague-Dawley (SD) rats with lipopolysaccharide (LPS)^13^ or zymosan^14^. Interestingly, these offspring developed moderate cardiac remodeling and fibrosis when elder than 8 months old^15^,^16^. However, the related mechanism remains elusive.

Considering modern high-paced tension induced heart diseases and unclarified mechanisms of prenatal inflammatory stimulus induced cardiac damage, we stimulated the adult offspring of LPS-treated mothers with 2 weeks of ISO treatment. We then assessed for the cardiac function, hypertrophy index, and degree of fibrosis. In this report, we found that prenatal exposure to inflammatory stimulus predisposed offspring to ISO-induced cardiac hypertrophy, fibrosis and myocardial contractility dysfunction in adulthood. Mechanically, pre-existed increased reactive oxygen species (ROS) levels and p38 activity produced a positive feedback to enhance ROS generation mainly through up-regulating nicotinamide adenine dinucleotide phosphate-oxidase (NADPH oxidase) expression in response to ISO stimulation. This in turn led to augmented ISO-induced cardiac damage. Hence, maternal inflammatory exposure has the potential to induce myocardium damage and accelerates heart injury in adult offspring when meeting with SNS activation or other risk factors through increased oxidative stress.

## Results

### Maternal inflammation exacerbates left-ventricular systolic dysfunction resulted from ISO stimulation in adult offspring

**Figure 1:**
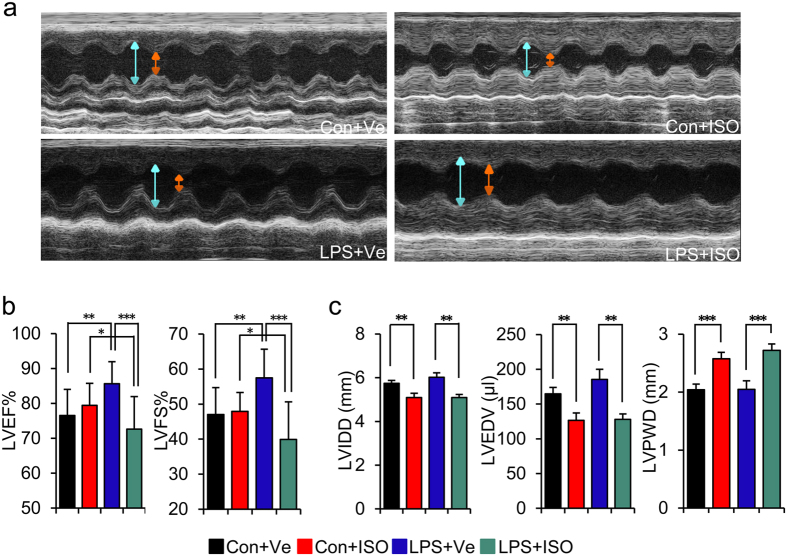
Maternal inflammation aggravates cardiac systolic dysfunction caused by 2 weeks of isoproterenol stimulation in adult offspring. Pregnant SD rats were administered intraperitoneally (i.p) with saline (Control group) or LPS (0.79 mg/kg, LPS group) at gestational day (GD) 8, 10 and 12. Offspring from both control and LPS group at the age of 20 weeks were treated with vehicle (Con + Ve group and LPS + Ve group, respectively) or ISO (Con + ISO group and LPS + ISO group, respectively) for 2 weeks. (**a**) At the end of ISO treatment, cardiac function was assessed by echocardiography. Representative photo-micro- graphs from M-mode echocardiography. (**b**) Left ventricular systolic function index. LVEF%: left ventricular ejection fraction; LVFS%: left ventricular fractional shortening. (**c**) Left ventricular diastolic function index. LVIDD: Left ventricular end diastolic internal dimension; LVEDV: Left ventricular end diastolic volume; LVPWD: Left ventricular end diastolic posterior wall dimension. Error bar represents S.D. n = 15 offspring in Con + Ve and LPS + Ve group, n = 11 offspring in Con + ISO and LPS + ISO group. *p < 0.05, **p < 0.01, ***p < 0.001 and ^#^*p* < 0.05 denote the statistical comparison between the two marked treatment groups, respectively. Two-way ANOVA.

We have previously found that offspring of LPS-treated mothers exhibited moderate increased collagen synthesis but without any changes of cardiac function at a younger stage^15^. These offspring gradually acquired the phenotype of left ventricle (LV) hypertrophy and diastolic dysfunction at 8 months of age^16^. These findings indicated that prenatal inflammatory exposure may predispose offspring to cardiac damage when challenging with a second cardiovascular risk factor after birth. We tested the cardiac function in response to the ISO stimulation as a second risk factor challenge in offspring of LPS-treated mothers to test the above hypothesis. We stimulated offspring with low dose ISO (5 mg/kg/day) for 2 weeks starting at the age of 20 weeks, followed by the assessment of cardiac function by echocardiography (Fig. 1 and Supplementary Table s2). Representative examples of M-Mode echocardiography are shown in Fig. 1a. We found pre-existed enhanced index of left ventricular ejection fraction (LVEF%) and left ventricular fractional shortening (LVFS%) in offspring of LPS-treated mothers as compared to those in control (**LPS + Ve** vs. **Con + Ve**, *p* < 0.01) (Fig. 1b). Combined with our previous finding of left ventricle diastolic dysfunction at an elder age^16^, this suggests a compensatory enhancement of myocardium contractility existed in adult offspring of LPS-treated mothers at a younger age. Low dose ISO treatment only slightly affected the cardiac contractility function, as evident by the decreased index of LVEF% and LVFS% without reaching statistical significance in control offspring (**Con + ISO** vs. **Con + Ve**, *p* > 0.05) (Fig. 1b). Our findings were consistent with previously reported literature^17^,^18^. However, ISO challenge significantly decreased the index of LVEF% and LVFS% in offspring of LPS-treated mothers as compared to those with vehicle treated alone (**LPS + ISO** vs. **LPS + Ve**, *p* < 0.001) or control offspring with ISO treatment (**LPS + ISO** vs. **Con + ISO**, *p* < 0.05) (Fig. 1b). In regard to cardiac diastolic function, ISO treatment decreased left ventricular end diastolic internal dimension (LVIDD) and left ventricular end diastolic volume (LVEDV) (**Con + ISO** vs. **Con + Ve**, *p* < 0.01; LPS + ISO vs. LPS + Ve, *p* < 0.01), but increased left ventricular end diastolic posterior wall dimension (LVPWD) (**Con + ISO** vs. **Con + Ve**, *p* < 0.001; **LPS + ISO** vs. **LPS + Ve**, *p* < 0.001) in both offspring of LPS-treated mothers and that of control (Fig. 1c). Furthermore, the diastolic function index had no difference between offspring of LPS-treated mothers and that of control in challenge of ISO (**LPS + ISO** vs. **Con + ISO**, *p* > 0.05) (Fig. 1c). Previous studies have demonstrated gender difference in β-AR system response^19^,^20^, we also used one-way ANOVA analysis to analyze our above data in female or male offspring, separately. In control offspring, there was a declined trend in LVEF% and LVFS% in male offspring but not in female offspring after ISO treatment (Supplementary Fig. s1a,b, Table s3), which is consistent with previous other findings^19^,^20^. Interestingly, female offspring of LPS-treated mothers seems to be more sensitive to ISO challenge than that of male offspring, as evidenced by the p value between LPS + ISO and LPS + Ve is smaller for female offspring than that in male offspring (LPS + ISO vs. LPS + Ve, *p* < 0.01 and *p* < 0.05 for female and male offspring, respectively) (Supplementary Fig. s1 and Table s3). However, it showed no gender difference in LVEF% and LVFS% by two-way ANOVA analysis with ignoring gender (Supplementary Table s2, Gender, *p* > 0.05). All above reported data suggested that prenatal inflammatory exposure predisposes adult offspring to the cardiac systolic dysfunction with the challenge of cardiac risk factors without any gender difference.

### Prenatal inflammation exposure deteriorated ISO induced cardiac hypertrophy in adult offspring

**Figure 2:**
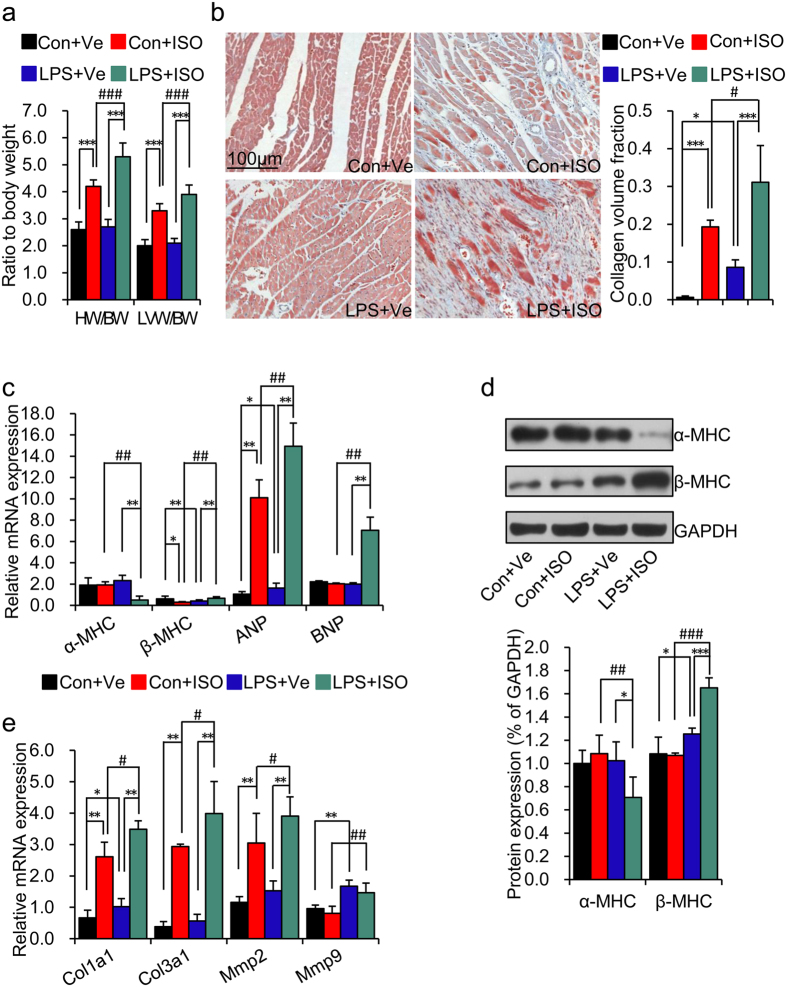
Prenatal inflammation exposure predisposes to isoproterenol-induced cardiac hypertrophy and myocardial fibrosis in adult offspring. Offspring were treated as describe in Fig. 1. (**a**) The index of heart weight to body weight ratio (HW/BW) and Left Ventricular Mass Index (LVW/BW) in offspring. n = 8 offspring in each group. (**b**) Representative images of Masson trichrome staining of LV sections. Myocardial fibrosis was quantified as the collagen volume fraction (CVF). Fibrous collagen: blue; myocyte: Red; scale bar = 100 um, 200×. n = 4 offspring per group. (**c**) The mRNA levels of α-myosin heavy chain (*α-MHC*), β-myosin heavy chain (*β-MHC*), atrial natriuretic peptide (*ANP*), and brain natriuretic peptide (*BNP*) were determined by real-time RT-PCR. *β-actin* was taken as internal control. n = 5 offspring in each group. (**d**) The protein expressions of α-MHC and β-MHC were determined by immunoblotting in left ventricle. Representative plots in each group and statistical data of relative densitometry, normalized by GAPDH, are shown. n = 5 offspring in each group. (**e**) The mRNA levels of myocardial fibrosis marker collagen type I (*Col1a1*), collagen type III (*Col3a1*), matrix metalloproteinases 2 (*Mmp2*) and *Mmp9* were determined by real-time RT-PCR. *β-actin* was taken as internal control. n = 5 offspring in each group. Error bar represents S.D. *p < 0.05, **p < 0.01, ***p < 0.001, ^#^*p* < 0.05, ^##^*p* < 0.01 and ^###^*p* < 0.001 denote the statistical comparison between the two marked treatment groups, respectively. Two-way ANOVA.

ISO induced cardiac hypertrophy and myocardial fibrosis are the classic pathophysiological bases of progressive cardiac dysfunction^21^. As such, we next determined whether aggravated cardiac hypertrophy and myocardial fibrosis existed in offspring of LPS-treated mothers after 2 weeks of ISO treatment. ISO treatment significantly increased the index of heart weight to body weight ratio (HW/BW) and left ventricular mass (LVW/BW) in offspring of both control and LPS-treated mothers (**Con + ISO** vs. **Con + Ve**, *p* < 0.001; **LPS + ISO** vs. **LPS + Ve**, *p* < 0.001) (Fig. 2a). Furthermore, the index of HW/BW and LVW/BW was significantly higher in offspring of LPS-treated mothers with ISO treatment as compared to that in control offspring with ISO treatment (**LPS + ISO** vs. **Con + ISO**, *p* < 0.001) (Fig. 2a). We next determined the myocardial fibrosis *via* Masson trichrome staining. Consistent with the result of systolic function, slightly increased myocardial fibrosis existed in offspring of LPS-treated mothers without ISO challenge (**LPS + Ve** vs. **Con + Ve**, *p* < 0.05) (Fig. 2b). We also showed that ISO treatment induced myocardial fibrosis in offspring of both control and LPS-treated mothers (**Con + ISO** vs. **Con + Ve**, *p* < 0.001; **LPS + ISO** vs. **LPS + Ve**, *p* < 0.001) (Fig. 2b). Moreover, higher levels of myocardial fibrosis existed in offspring of LPS-treated mothers after ISO treatment when compared to that in control offspring (**LPS + ISO** vs. **Con + ISO**, *p* < 0.05); (Fig. 2b).

To find more evidence of enhanced cardiac hypertrophy and myocardial fibrosis in offspring of LPS-treated mothers after ISO treatment, we also measured the gene expressions that related to cardiac hypertrophy and myocardial fibrosis, such as α-myosin heavy chain (*α-MHC*), β-myosin heavy chain (*β-MHC*), atrial natriuretic peptide (*ANP*), brain natriuretic peptide (*BNP*), collagen type I alpha 1 (*Col1a1*), collagen type III alpha 1 (*Col3a1*). matrix metalloproteinases 2 (*Mmp2*) and matrix metalloproteinases 9 (*Mmp9*)^22^. 2 weeks of ISO treatment significantly up-regulated *β-MHC*, *ANP* and *BNP* expression by 1.7, 29.0 and 3.5-fold respectively, and down-regulated *α-MHC* expression by 5.0-fold in offspring of LPS-treated mothers (**LPS + ISO** vs. **LPS + Ve**, *p* < 0.01) (Fig. 2c). The increased ratio of *α-MHC/β-MHC* mRNA expression, which is considered to be index of producing more cardiac output in heart^23^, was even decreased by 14-fold in offspring of LPS-treated mothers after ISO treatment (**LPS** + **ISO** vs. **LPS + Ve**, *p* < 0.01; Supplementary Fig. s2a). However, there was only a slightly changes in control offspring after ISO treatment except for an increased *ANP* mRNA expression (**Con + ISO** vs. **Con + Ve**, *p* < 0.01) (Fig. 2c). Furthermore, there were also significant changes in the above-mentioned data between control offspring and offspring of LPS-treated mothers after ISO treatment (**LPS + ISO** vs. **Con + ISO**, *p* < 0.01) (Fig. 2c). The protein level of α-MHC and β-MHC showed the similar trend as the mRNA expression showed in Fig. 2c (Fig. 2d and Supplementary Fig. s2b). Interestingly, we also found increased *α-MHC/β-MHC* ratio and *ANP* mRNA expression by 2.3 and 1.6-fold, respectively, in offspring of LPS-treated mothers as compared to that in control offspring (**LPS + Ve** vs. **Con + Ve**, *p* < 0.01 and 0.05, respectively). The mRNA expression of *Col1a1* and *Mmp9* was higher in offspring of LPS-treated mothers as compared to that in control offspring (**LPS + Ve** vs. **Con + Ve**, *p* < 0.05), while *Col3a1* and *Mmp2* displayed the similar trend (Fig. 2e). After 2 weeks of ISO treatment, the mRNA expression of *Col1a1*, *Col3a1* and *Mmp2* expression was significantly increased in offspring of both control and LPS-treated mothers (**Con + ISO** vs. **Con + Ve**, *p* < 0.01; **LPS + ISO** vs. **LPS + Ve**, *p* < 0.01) (Fig. 2e). In addition, statistical significance was also achieved between the offspring of both control and LPS-treated mothers after ISO treatment (**LPS + ISO** vs. **Con + ISO**, *p* < 0.05) (Fig. 2e).

All reported data above suggested that a compensatory cardiac hypertrophy exists in offspring of LPS-treated mothers; however, this compensatory effect soon proceeds to a decompensation stage whereas control offspring still have enough compensatory capacity response to a risk factor of cardiovascular diseases as a second hit. This might be a critical reason implicated in the increased susceptibility of cardiac damage during a second hit by cardiovascular risk factors. However, the exact mechanisms underlying this effect are largely unknown.

### Imbalance of ROS generation and elimination augments myocardial injury after ISO treatment in offspring of LPS-treated mothers

**Figure 3:**
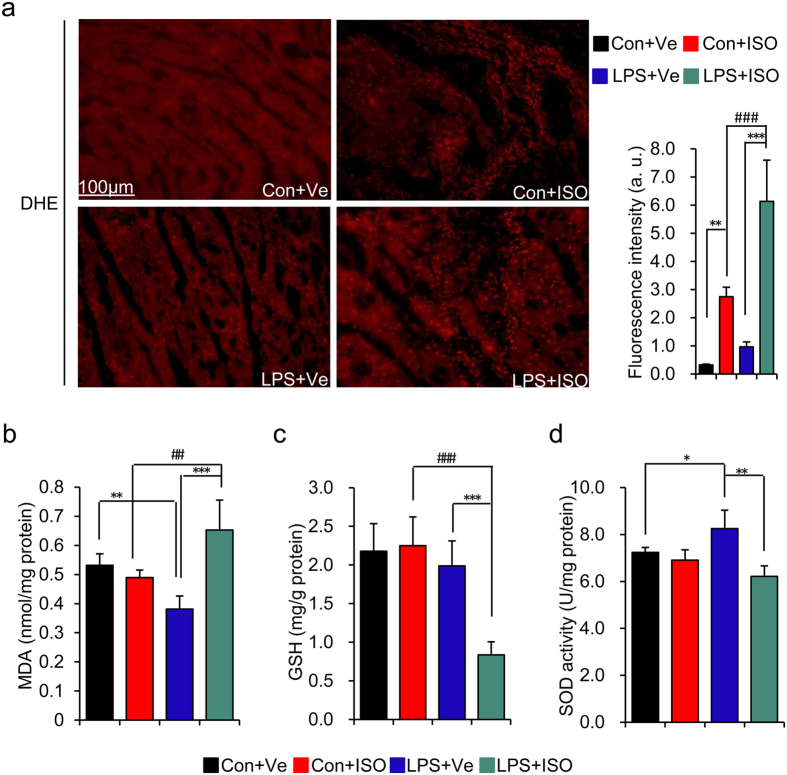
Offspring from LPS-treated mothers displays exaggerated oxidative damage after isoproterenol treatment. Offspring were treated as describe in Fig. 1. (**a**) ROS production was assessed by staining with dihydroethidium (DHE, red fluorescence) in fresh frozen section of left ventricle. Representative pictures from each group were shown (left panel) and the value of DHE fluorescence was quantified using Image J software (right panel). Scale bar = 100 um, 200×. n = 4 offspring in each group. (**b–d**) The level of malondialdehyde (MDA) (**b**), glutathione (GSH) (**c**) and antioxidants superoxide dismutase (SOD) activity (**d**) in LV tissue homogenates were quantified. n = 5 offspring in each group. Error bar represents S.D. *p < 0.05, **p < 0.01, ***p < 0.001, ^#^*p* < 0.05, ^##^*p* < 0.01 and ^###^*p* < 0.001 denote the statistical comparison between the two marked treatment groups, respectively. Two-way ANOVA.

It is well accepted that ROS generation is a common response in cells exposed to stressors and is involved in a variety of cellular signaling pathways, acting as second messengers. Mounting evidence has strongly implicated ROS activation in the genesis of cardiac hypertrophy and myocardial fibrosis, like matrix metalloproteinases (MMPs) and leads to the myocardial matrix remodeling^24^. This prompted us to investigate whether there existed an enhanced ROS generation in heart from offspring of LPS-treated mothers and also with ISO treatment. Dihydroethidium (DHE) staining showed that the ROS level was slightly higher in offspring of LPS-treated mothers than that in control offspring. Although ISO stimulation increased ROS level in both offspring of control and LPS-treated mothers, the ROS level was significantly higher in offspring of LPS-treated mothers than that of control offspring after ISO treatment (Fig. 3a). To find more direct evidence for augmented tissue oxidative damage in offspring of LPS-treated mothers after ISO treatment, we also assessed the biochemical markers of oxidative stress, including malondialdehyde (MDA) level, glutathione (GSH) level and antioxidants superoxide dismutase (SOD) activity in left ventricular. MDA is the product of polyunsaturated fatty acid peroxidation in cells, which is an important marker of oxidative damage in tissues^25^. Our data showed that the level of MDA was decreased in offspring of LPS-treated mothers as compared to that of control mothers (LPS + Ve vs. Con + Ve, *p* < 0.01) (Fig. 3b). After ISO treatment, MDA level was significantly increased in offspring of LPS-treated mothers (LPS + ISO vs. LPS + Ve, *p* < 0.001), which was also significantly higher than that of control offspring with ISO treatment (LPS + ISO vs. Con + ISO, *p* < 0.01) (Fig. 3b). We next determined the level of GSH in LV tissue, which is the most abundant non-protein thiol that defends against oxidative stress and also a key determinant of redox signaling^26^. Consistent with the finding of MDA level in LV, the level of GSH was significantly decreased after ISO treatment, as compared to that of vehicle-treated offspring of LPS-treated mothers or ISO treated control offspring (LPS + ISO vs. LPS + Ve, *p* < 0.001; LPS + ISO vs. Con + ISO, *p* < 0.001) (Fig. 3c). The data of SOD activity showed that offspring of LPS-treated mothers showed a higher level of SOD activity as compared to that of control offspring (LPS + Ve vs. Con + Ve, *p* < 0.01), whereas ISO treatment markedly decreased its SOD activity in offspring of LPS-treated mothers compared to that of vehicle-treated offspring of LPS-treated mothers or control offspring with ISO treatment (LPS + ISO vs. LPS + Ve, *p* < 0.001; LPS + ISO vs. Con + ISO, *p* < 0.001) (Fig. 3d). All the above results demonstrated that the ROS activation preexists in offspring of LPS-treated mothers and a second hit can cause an obvious tissue oxidative damage.

**Figure 4:**
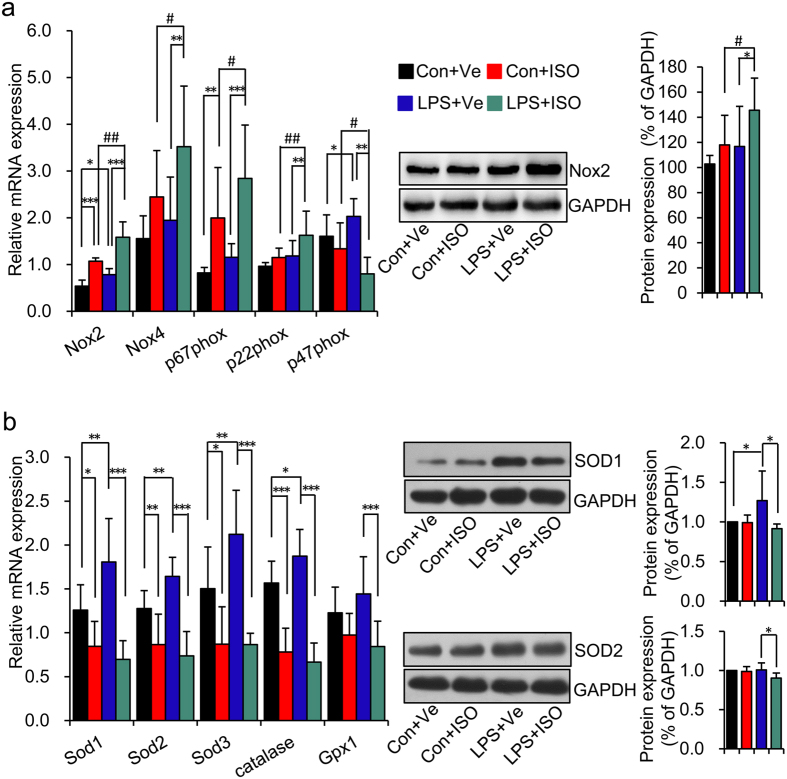
Increased NADPH oxidase contributes to an imbalance of ROS generation and elimination in offspring from LPS-treated mothers after isoproterenol treatment. Offspring were treated as describe in Fig. 1. (**a**) The mRNA levels and protein levels of ROS generation related genes (NADPH oxidase subunit, such as *Nox2, Nox4, p67*^*phox*^, *p47*^*phox*^ and *p22*^*phox*^) in left ventricle were determined by real-time RT-PCR or immunoblotting, respectively. n = 5 offspring in each group. (**b**) The mRNA levels and protein levels of ROS elimination related genes (antioxidant enzymes, such as *Sod1, Sod2, Sod3, catalase* and *Gpx1*) in left ventricle were determined by real-time RT-PCR and immunoblotting, respectively. *β-actin* was taken as internal control in real-time RT-PCR. Representative plots in each group and statistical data of relative densitometry, normalized by GAPDH, are shown. n = 5 offspring in each group. Error bar represents S.D. *p < 0.05, **p < 0.01, ***p < 0.001, ^#^*p* < 0.05, ^##^*p* < 0.01 and ^###^*p* < 0.001 denote the statistical comparison between the two marked treatment groups, respectively. Two-way ANOVA.

To further explore the reason for oxidative damage in offspring of LPS-treated mothers with ISO treatment, we determined the signal pathways for ROS generation and clearance. We first assessed the mRNA expression of the general enzyme for intracellular ROS generation, NADPH oxidase, which is a professional ROS producer by one-electron reduction of oxygen using reduced NADPH as the electron donor^27^. In the heart, the NADPH oxidase contains membrane-bound subunits Nox2, Nox4 and p22^phox^, as well as cytosolic regulatory subunits p67^phox^ and p47^phox^, which can be activated by various cellular stressors, such as chemical factors, physical challenges, stress-related humoral or neural factors^27^. Increased mRNA expression of *Nox2* and *p47*^*phox*^ was found in heart of offspring from LPS-treated mothers, as compared to that in control (**LPS + Ve** vs. **Con + Ve**, *p* < 0.05) (Fig. 4a, left panel), while the mRNA expression of *Nox4, p67*^*phox*^ and *p22*^*phox*^ also showed the similar trend though there was no statistical significance (Fig. 4a, left panel). 2 weeks of ISO challenge led to increased mRNA expression of *Nox2* and p67^*phox*^ in the heart tissue of control offspring (**Con + ISO** vs. **Con + Ve**, *p* < 0.01) (Fig. 4a, left panel), which is consistent with previous research in this area^28^. Consistent with our above finding in ROS, the mRNA expression of above mentioned subunits of NADPH oxidase in the heart of offspring from LPS-treated mothers with ISO treatment was significantly higher than that with vehicle treatment or control offspring with ISO treatment, respectively (**LPS + ISO** vs. **LPS + Ve**, *p* < 0.01; **Con + ISO** vs. **LPS + ISO**, *p* < 0.01 or 0.05) (Fig. 4a, left panel). Interestingly, ISO treatment led to reduced *p47*^*phox*^ mRNA expression by 2.8-fold in offspring of LPS-treated mothers (**LPS + ISO** vs. **LPS + Ve**, *p* < 0.01), which was also lower than that in control offspring with ISO treatment (**LPS + ISO** vs. **Con + ISO**, *p* < 0.05) (Fig. 4a, left panel). This result indicated that impaired *p47*^*phox*^ expression may also participate in exaggerated cardiac damage independent of its capacity in regulating NADPH oxidase activity^29^. We also confirmed the protein expression of Nox2 by immunoblotting, which showed the similar trend as its mRNA level (Fig. 4a, right panel). These data above demonstrated that prenatal exposure to inflammation disperses the expression of several NADPH oxidase subunits, whereas a second hit of cardiovascular risk factor augments this action.

We next determined the gene expression response for ROS clearance, such as superoxide ion (O_2_^.−^) scavenger copper-zinc SOD (*Sod1*), manganese SOD (*Sod2*) and extracellular SOD (*Sod3*), as well as hydrogen peroxide (H_2_O_2_) scavenger glutathione peroxidase (*Gpx1*) and *catalase*^30^. In early stages of tissue injury, homeostatic up-regulation of these antioxidant enzymes in response to increased free radicals acts as a protective system to prevent tissue damage. However, this compensation ceases as soon as free radicals reach to and maintain at the higher levels^31^. Consistent with the previous finding of pre-existing higher level of cardiac ROS in offspring of LPS-treated mothers (Fig. 4a), the mRNA expression of *Sod1*, *Sod2*, *Sod3* and *catalase* in offspring of LPS-treated mothers was significantly higher than that in control offspring (**LPS + Ve** vs. **Con + Ve**, *p* < 0.01 or 0.05) (Fig. 4b, left panel), though *Gpx1* only showed tendency of increment (Fig. 4b, left panel). However, the mRNA expression of above genes was significantly down-regulated in the heart tissue in offspring of LPS-treated mothers after 2 weeks of ISO treatment, but no difference with control offspring (**LPS + ISO** vs. **LPS + Ve**, *p* < 0.001; **LPS + ISO** vs. **Con + ISO**, *p* > 0.05) (Fig. 4b, left panel). However, the expression of above antioxidant enzymes was reduced to a greater extent after ISO treatment in offspring of LPS-treated mothers compared to that in control offspring (Supplementary Fig. s3). The protein levels of SOD1 and SOD2 were also consistent with their transcript levels (Fig. 4b, right panel). These results indicated aggravated oxidative damage in offspring of LPS-treated mothers with ISO challenge is mainly attributed to increased ROS generation via augmented elevated expression of NADPH oxidases.

**Figure 5:**
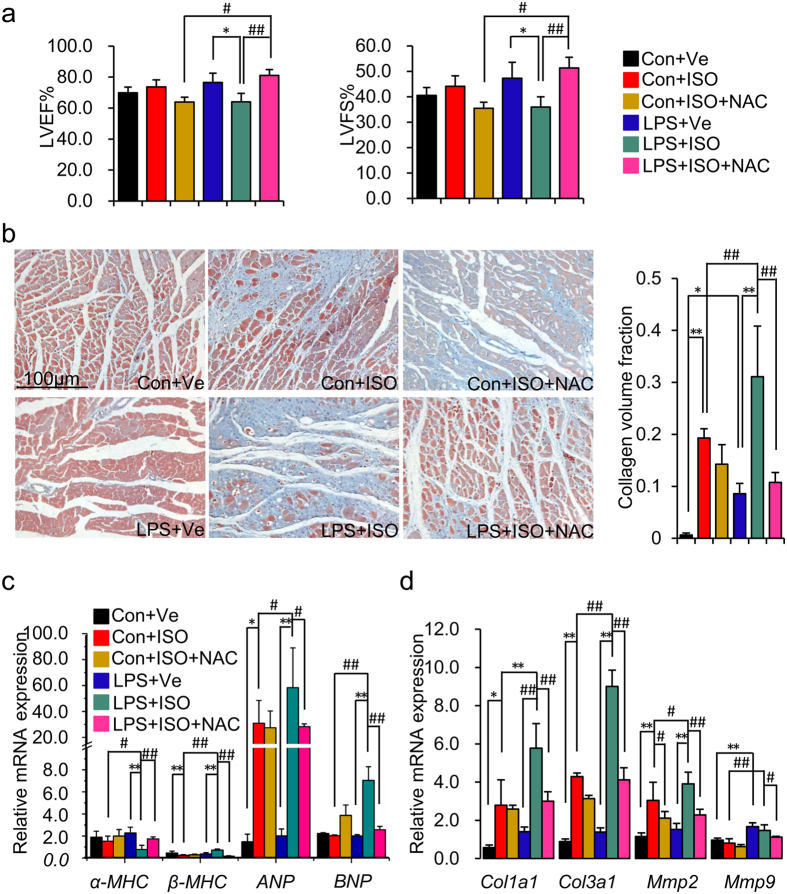
Antioxidant N-acetyl-L-cysteine (NAC) reverses myocardial damage in offspring of LPS-treated mothers after isoproterenol treatment. Offspring were treated as describe in Fig. 1 by adding NAC treatment simultaneously with ISO treatment for 2 weeks in both Con + ISO and LPS + ISO group, defined as Con + ISO + NAC and LPS + ISO + NAC group, respectively. (**a**) Representative echocardiography of LVEF%, LVFS%. n = 6 offspring in each group. (**b**) Representative images of Masson trichrome staining of LV sections. Fibrous collagen: blue; myocyte: Red; scale bar = 100 um, 200×. n = 4 offspring in each group. Representative pictures from each group were shown (left panel) and the value of collagen volume fraction was quantified with an image analysis system (right panel). (**c,d**) The mRNA levels of *α-MHC, β-MHC, ANP, BNP* (**c**) and *Col1a1*, *Col3a1*, *Mmp2*, *Mmp9* (**d**) were determined by real-time RT-PCR. *β-actin* was taken as internal control. n = 5 offspring in each group. Error bar represents S.D. *p < 0.05, **p < 0.01, ^#^*p* < 0.05, and ^##^*p* < 0.01 denote the statistical comparison between the two marked treatment groups, respectively. Two-way ANOVA.

These above findings of impaired balance of ROS generation and clearance in offspring of LPS-treated mothers in response to ISO treatment prompted us to further explore the physiological role in ROS activation in augmenting heart damages induced by ISO through administration of N-acetyl-L-cysteine (NAC), which is a glutathione (GSH) precursor and acts as direct antioxidant^32^. Echocardiography showed that NAC administration improved the index of LVEF% and LVFS% and prevented myocardial fibrosis, caused by 2 weeks of ISO treatment (**LPS + ISO + NAC** vs. **LPS + ISO**, *p* < 0.01) (Fig. 5a,b). However, there seems no obvious effect of NAC on control offspring (Fig. 5a,b). Consistent with the protective effect of NAC on cardiac pathophysiological changes, real-time RT-PCR analysis of the mRNA expressions that related to cardiac hypertrophy and myocardial fibrosis, such as *α-MHC, β-MHC, ANP, BNP, Col1a1, Col3a1, Mmp2,* and *Mmp9,* also showed the similar trend (Fig. 5c,5d).

We also determined the role of NAC on the gene expression that related to ROS generation and elimination. NAC reversed the increased mRNA expression of *Nox2*, *Nox4*, *p67*^*phox*^ and *p22*^*phox*^ in heart tissue of offspring from LPS-treated mothers with ISO treatment (**LPS + ISO + NAC** vs. **LPS + ISO**, *p* < 0.01 or 0.05) (Supplementary Fig. s4a). NAC also improved the mRNA expression of *Sod1*, *Sod2*, *Sod3*, and *Gpx1* in the heart tissue of offspring from LPS-treated mothers with ISO challenge (**LPS + ISO + NAC** vs. **LPS + ISO**, *p* < 0.01 or 0.05) (Supplementary Fig. s4b). Once the excessive ROS was scavenged, the up-regulation of anti-oxidant enzymes mRNA expression appeared again, which further confirmed our idea that prenatal exposure to inflammation leads to increased ROS generation via up-regulating the expression of NADPH oxidases accompanied by the compensatory increased expression of anti-oxidant enzymes in offspring at resting state. Once suffering from a second hit, the continued elevated expression of NADPH oxidases and ROS level disperse the balance of oxidant and antioxidant capacity, which soon switch on the process of oxidative damage on heart by excessive free radicals.

### p38 MAPK activation participates in increased ROS level mediating aggravated heart dysfunction caused by ISO challenge in offspring of LPS-treated mothers

**Figure 6:**
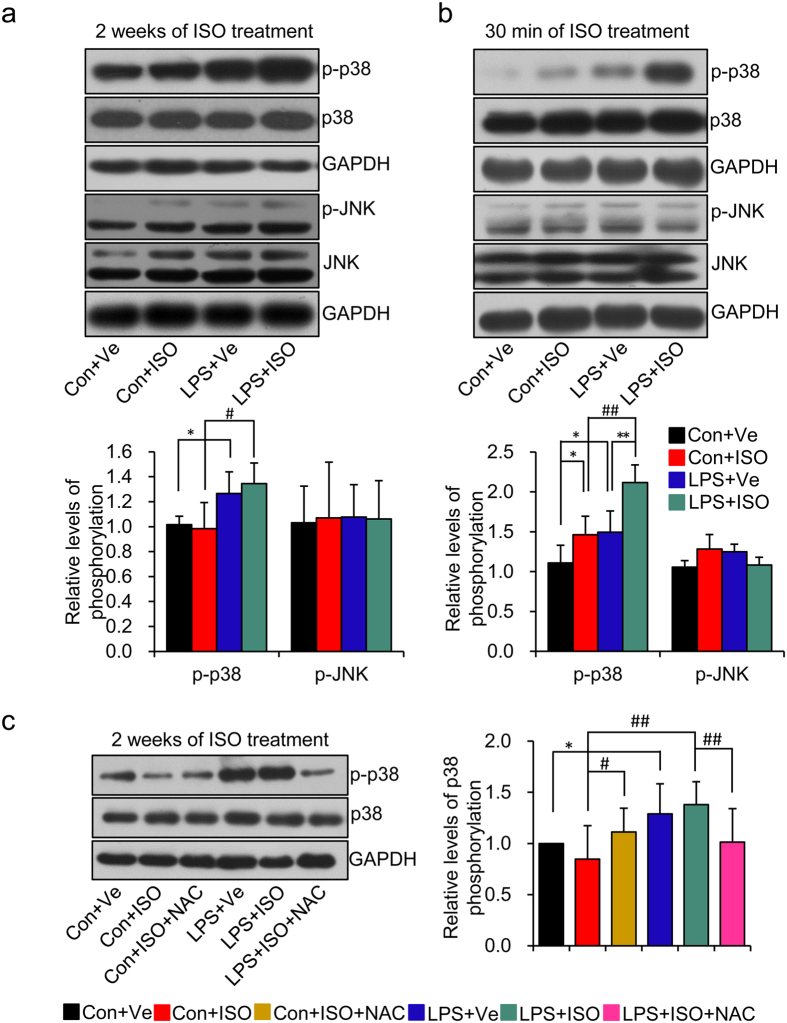
ROS-dependent p38 activation is the main sensor response to amplified oxidative stress in offspring of LPS-treated mothers after isoproterenol treatment. (**a,b**) Offspring at the age of 20 weeks were treated with ISO for 2 weeks (**a**) or 30 min (**b**) as described in Fig. 1 and the protein expressions of phosphorylated (p)-p38, p38, p-JNK and JNK in left ventricle were assessed by immunoblotting. n = 6 offspring in each group for a and b. (**c**) Offspring were treated as described in Fig. 5 and the protein levels of p-p38 and p38 were determined by immunoblotting. Representative plots in each group and statistical data of relative densitometry, normalized by GAPDH, are shown. n = 5 offspring in each group. *p < 0.05, **p < 0.01, ^#^*p* < 0.05 and ^##^*p* < 0.01 denote the statistical comparison between the two marked treatment groups, respectively. Two-way ANOVA.

In the heart, ROS can directly or indirectly activate various downstream signaling pathways in response to extracellular or intracellular factors that mediate the hypertrophic growth^27^. This includes mitogen-activated protein kinase (MAPK) signal pathway, mainly p38 and c-jun N-terminal kinase (JNK) MAPK, which are known as the major intracellular stress sensors^33^. Thus, we next explored whether these signaling pathways participate in the exaggerated heart damages caused by ISO challenge in offspring of LPS-treated mothers and its relationship with ROS activation. At the end of ISO treatment, the level of p38 but not JNK phosphorylation was significant higher in offspring of LPS-treated mothers than that in control offspring irrespective of ISO treatment (**LPS + Ve** vs. **Con + Ve**, *p* < 0.05; **LPS + ISO** vs. **Con + ISO**, *p* < 0.05) (Fig. 6a). Unexpectedly, there was no differences of p38 or JNK phosphorylation level between ISO or vehicle treated offspring from both control and LPS-treated mothers, respectively (**Con + ISO** vs. **Con + Ve**, *p* > 0.05; **LPS + ISO** vs. **LPS + Ve**, *p* > 0.05) (Fig. 6a).

Previous reports identified that MAPKs were activated rapidly responding to ISO treatment but became exhausted if a persistent activating signal existed^34^. As such, this prompted us to further test the rapid reactivity of p38 or JNK MAPK activation in response to a short term ISO challenge. After 30 minutes of a one-time ISO injection (5 mg/kg, subcutaneous injections), obvious p38 phosphorylation was observed in the heart tissue of both offspring of control and LPS-treated mothers (**Con + ISO** vs. **Con + Ve**, *p* < 0.05; **LPS + ISO** vs. **LPS + Ve**, *p* < 0.01) (Fig. 6b). However, it was much higher in offspring of LPS-treated mothers with ISO treatment (**LPS + ISO** vs. **Con + ISO**, *p* < 0.01) (Fig. 6b). Interestingly, we did not observe any significant difference of JNK phosphorylation in this acute model of ISO treatment (Fig. 6b). Furthermore, NAC reversed the increased p38 phosphorylation level in offspring of LPS-treated mothers after 2 weeks of ISO treatment (**LPS + ISO + NAC** vs. **LPS + ISO**, *p* < 0.01) (Fig. 6c). These data indicated that the rapid over-activation of p38 MAPK, caused by ISO stimulation, in offspring of LPS-treated mothers plays a critical role in augmented heart damage when responding to secondary risk factors and is also ROS dependent.

**Figure 7:**
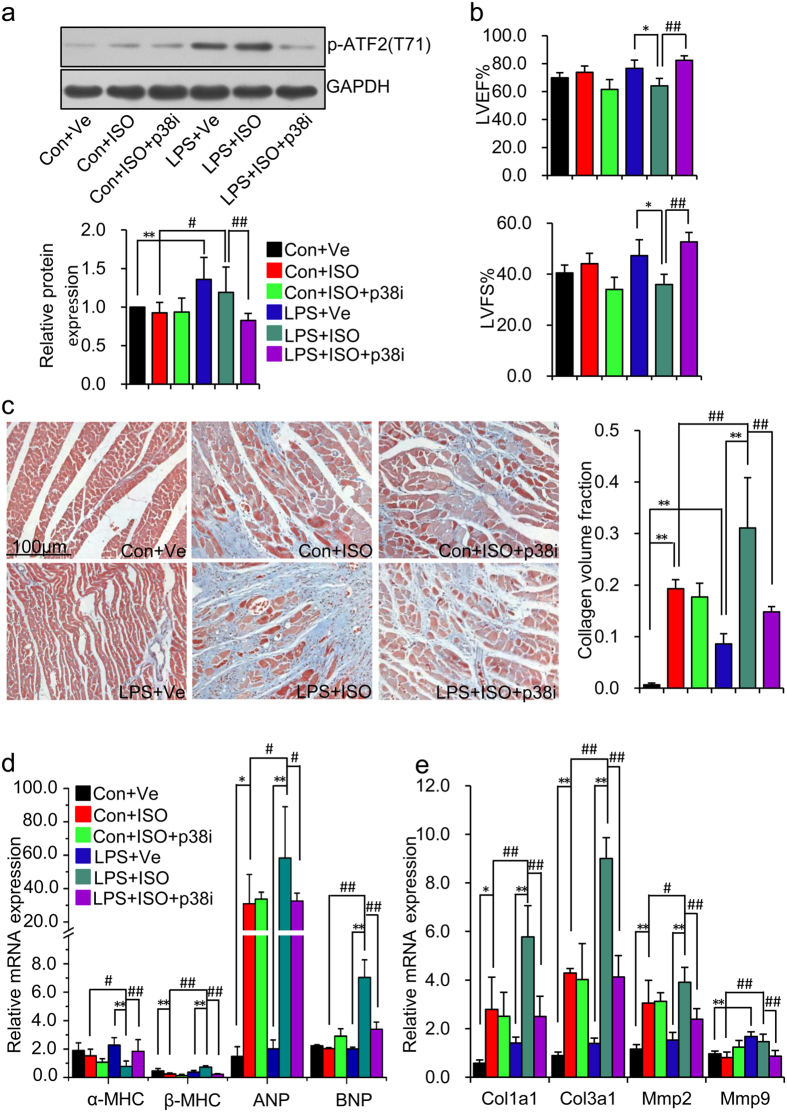
p38 MAPK activation augments isoproterenol-induced cardiac contraction decline and myocardial fibrosis in prenatal inflammation exposure offspring. Offspring were treated as describe in Fig. 1 by adding p38 inhibitor SB202190 treatment simultaneously with ISO treatment for 2 weeks in both Con + ISO and LPS + ISO group, defined as Con + ISO + p38i and LPS + ISO + p38i group, respectively. (**a**) p-ATF2, the downstream targets of p38 activation, was determined by immunoblotting. Representative plots in each group and statistical data of relative densitometry, normalized by GAPDH, are shown. n = 5 offspring in each group. (**b**) Representative echocardiography of LVEF%, LVFS%. n = 6 offspring in each group. (**c**) Representative images of Masson trichrome staining of LV sections (left panel). Myocardial fibrosis was quantified as CVF (right panel). Fibrous collagen: blue; myocyte: red; scale bar = 100 um, 200 × . n = 4 rats per group. (**d,e**) The mRNA levels of *α-MHC, β-MHC, ANP, BNP* (**d**) and *Col1a1*, *Col3a1*, *Mmp2*, *Mmp9* (**e**) in left ventricle were determined by real-time RT-PCR. *β-actin* was taken as internal control. n = 5 offspring in each group. Error bar represents S.D. *p < 0.05, **p < 0.01, ^#^*p* < 0.05, and ^##^*p* < 0.01 denote the statistical comparison between the two marked treatment groups, respectively. Two-way ANOVA.

To further explore the physiological role of p38 activation in mediating deteriorative heart damage in offspring of LPS-treated mothers in response to ISO challenge, we used a p38 specific inhibitor that had no effect on the activity of JNK MAPK signal pathway^35^, along with ISO treatment. Inhibition of p38 activity by (4-Fluorophenyl)-2-(4-hydroxylphenyl)-5-(4-pyridyl)-1H-imidazole (SB202190) was confirmed by the down-regulated phosphorylation of activating transcription factor-2 (ATF-2)^36^, the downstream molecule of p38 MAPK (**LPS + ISO + p38i** vs. **LPS + ISO**, *p* < 0.01) (Fig. 7a). Inhibition of p38 activation improved the myocardium output (increased index of LVEF% and LVFS%) in offspring of LPS-treated mothers with ISO treatment, whereas showed no obvious side effect on control offspring (**LPS + ISO + p38i** vs. **LPS + ISO**, *p* < 0.01; **Con + ISO + p38i** vs. **Con + ISO**, *p* > 0.05) (Fig. 7b). Masson staining also showed that p38 inhibition reversed myocardial fibrosis caused by ISO treatment in offspring of LPS-treated mothers (**LPS + ISO + p38i** vs. **LPS + ISO**, *p* < 0.01) (Fig. 7c). Consistent with its protective role on cardiac functions, p38 inhibition also reversed the mRNA expression of *α-MHC*, *β-MHC*, *ANP*, *BNP*, *Col1a1*, *Col3a1*, *Mmp2* and *Mmp9* in offspring of LPS-treated mothers after ISO treatment (Fig. 7d,e). This data demonstrated that high susceptibility to myocardial injury caused by the stressor is mainly mediated by ROS-dependent activation of p38 signal pathway in offspring of LPS-treated mothers.

### p38 MAPK activation amplified the imbalance of ROS generation and elimination in offspring of LPS-treated mothers with ISO challenge via up-regulating NADPH oxidase

**Figure 8:**
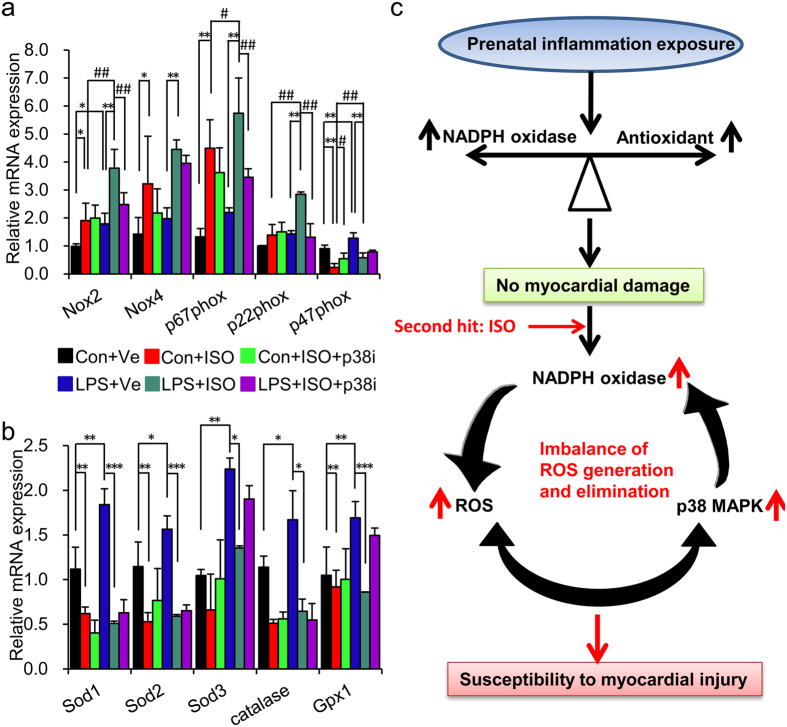
p38 activation leads to augmented oxidative stress in offspring of LPS-treated mothers after isoproterenol treatment and schematic illustration of the current study. Offspring were treated as described in Fig. 6. (**a,b**) The mRNA levels of *Nox2, Nox4, p67*^*phox*^*, p47*^*phox*^*, p22*^*phox*^ (**a**) and *Sod1, Sod2, Sod3*, *catalase*, *Gpx1* (**b**) in left ventricle were determined by real-time RT-PCR. *β-actin* was taken as internal control. n = 5 offspring in each group. Error bar represents S.D. *p < 0.05, **p < 0.01, ***p < 0.001, ^#^*p* < 0.05 and ^##^*p* < 0.01 denote the statistical comparison between the two marked treatment groups, respectively. Two-way ANOVA. (**c**) Schematic illustration of the mechanisms responsible for the augmented myocardium damages in response to risk factor challenges in adult offspring of LPS-treated mothers. Prenatal inflammatory stimulation leads to augmented cardiac hypertrophy and fibrosis, even systolic dysfunction induced by ISO in adult offspring. The activated ROS-p38-ROS positive feedback loop results in imbalance of ROS generation and elimination might be the main reason that aggravates cardiac damages responding to a second risk factor.

Previous reports have proved that p38 MAPK activation is involved in vascular or fibroblast ROS production through influencing the expression of NADPH oxidase, such as *Nox2* and *p47*^*phox*^ 37,38. This motivated us to explore whether the positive feedback loop of ROS-p38 MAPK-ROS is involved in the higher susceptibility to cardiovascular risk factors in offspring of LPS-treated mothers. In this model, inhibiting p38 activation by SB202190, we found that the mRNA expression of *Nox2*, *p67*^*phox*^ and *p22*^*phox*^ was remarkably inhibited by SB202190 treatment in offspring of LPS-treated mothers with ISO challenge (**LPS + ISO + p38i** vs. **LPS + ISO**, *p* < 0.01) (Fig. 8a). However, SB202190 did not show any effect on the mRNA expression of *Sods, Gpx1* and *catalase* in offspring of LPS-treated mothers after ISO treatment (**LPS + ISO + p38i** vs. **LPS + ISO**, *p* > 0.05) (Fig. 8b). We also assessed the biochemical markers of oxidative stress, including MDA level, GSH level and SOD activity in left ventricular. The results showed that inhibition of p38 activation decreased MDA level (**LPS + ISO + p38i** vs. **LPS + ISO**, *p* < 0.01), but without any effect on SOD activity and GSH level (Supplementary Fig. s5a–c) in offspring of LPS-treated mothers with ISO treatment. All these data above demonstrated that p38 activation mediates up-regulated expression of NADPH oxidase subunits which is implicated in the positive feedback loop of ROS-p38 MAPK-ROS, and in turn aggravates the myocardial damages by promoting imbalance of ROS generation and elimination when exposed to a cardiovascular risk factor in offspring of LPS-treated mothers.

## Discussion

The major findings of this study are as follows: (i) Prenatal LPS exposure predisposes myocardial toxicity caused by ISO in adult offspring, characterized by decreased left ventricular systolic as well as increased cardiac hypertrophy and myocardial fibrosis. (ii) Mechanistically, pre-existed ROS activation via up-regulating NADPH oxidases expression accompanied by the self-compensatory up-regulation of anti-oxidant capacity in the heart tissue at resting state, which switches on imbalance of ROS generation and elimination on heart rapidly when suffering from a myocardial hypertrophic challenge in offspring of LPS-treated mothers. (iii) ROS-dependent p38 MAPK activation amplifies the ROS generation through a ROS-p38 MAPK-NADPH oxidase-ROS positive feedback loop, which in turn aggravates the offspring of LPS-treated mothers to heart damage when exposed to a myocardial hypertrophic challenge.

The incidence of CVD is dramatically increasing at earlier ages. More than 17% of deaths caused by CVD occurred < 60 years old in 2012^39^. The trigger of increasing CVD deaths in younger people is associated with adverse psychological stress, which might be closely related with SNS activation, and includes sedentary behavior, sleep insufficiency, depression and other attributable causes^3^. Growing evidences show that maternal adverse exposure increases susceptibility to cardiac diseases in adult offspring^12^,^40^,^41^,^42^,^43^. We previously found that intra-peritoneal injection of LPS led to rousted increased TNF-α and IL-6 levels in both maternal serum and placenta, immediately, while IL-6 increased significantly after 48 hours in embryo^44^. This demonstrated that LPS treatment on gestation days 8, 10 and 12 triggered immune responses in both pregnant rats and their embryos. The current study has demonstrated a new potential maternal risk factor of prenatal inflammatory exposure during pregnancy can potentiate cardiac damage in adulthood^11^,^12^. As such, this new risk factor may contribute to the trend of earlier onset of cardiovascular disease. In support of this, we have found offspring of prenatal LPS exposure have higher susceptibility to cardiac hypertrophy, myocardial fibrosis and decreased systolic function by challenge with 2 weeks of ISO treatment. Interestingly, others have reported that these pathogenic effects may not be enough on their own to cause the decline of cardiac contraction in normal rats^18^. ISO is believe to be a good activator of β-adrenergic receptors (βARs) in heart^7^ and over activation of the β-AR pathway may be responsible for cardiovascular adjustment to physical and psychological stress induced cardiomyopathy^45^,^46^. Thus, the combined effects of prenatal inflammatory exposure and postnatal high pace of life style or other stressors might be likely to have a profound impact on offspring’s cardiac diseases as they progress into adulthood. Previous reports suggests a greater decline in the contractile response to ISO in men as compared to women^19^,^20^, however, female offspring of prenatal inflammation treated mothers seems to be more sensitive to ISO challenge than that of male offspring though without statistical significance in our model. This indicates that prenatal inflammatory exposure may also affect female offspring’s estrogen responding system, which is an interesting research field warranting future investigation.

Imbalance of ROS generation and elimination is critical to myocardium injury in several cardiac diseases, which are characterized by the accumulation of excessive intracellular ROS with increased enzymatic sources of ROS, such as NADPH oxidases and decreased antioxidant enzymes^47^. Excessive intracellular ROS modifies various signaling kinases and activates transcription factors related to hypertrophy or fibrosis which in turn results in cardiac tissue damage. However, at the beginning of the compensatory stage, low level of ROS, also called ROS activation^48^, contributes to balance various stress adaptation by up-regulating the anti-oxidant system to antagonize the ROS generation system, which also acts as a second messenger to maintain cardiac output^49^. When the level of free radicals keeps on rising, the expression and activity of antioxidant enzymes may reach to the saturation and further suddenly declined, which mediates the tissue oxidative damage^31^. Consistent with this, our current study found that offspring of LPS-treated mothers showed a slightly increased level of ROS, increased mRNA levels of NADPH oxidase and anti-oxidant enzymes, increased SOD activity and decreased MDA level, consistent with compensatory enhanced systolic function in heart. However, at the end of ISO treatment, offspring from LPS-treated mothers had significantly higher ROS levels, increased mRNA levels of NADPH oxidases but dramatic decline of antioxidant enzymes expression together with augmented LV oxidative damage and decreased cardiac output. Furthermore, NAC partly reversed the mRNA expression of antioxidant enzymes and cardiac function after ISO treatment in offspring of LPS-treated mothers, which indicated that the main contribution of tissue oxidative damage caused by ISO treatment in offspring of LPS-treated mothers attributes to the augmented expression of ROS generation enzymes, especially NADPH oxidases. This suggests that increased expression of NADPH oxidase and ROS activation pre-exist in the heart of offspring from LPS-treated mothers, accompanies with compensatory up-regulating antioxidant capacity at resting state, which protects the heart from oxidative damages. Augmented increment of NADPH oxidases expression in response to postnatal risk factor re-stimulation in offspring of prenatal inflammation-treated mothers mainly contributes to exacerbate tissue oxidative damage and cardiac dysfunction.

In rodent models, it is proved that over-activation of the p38 MAPK signaling pathway worsens myocardial pathology, contractile function and electrophysiology in several cardiac diseases^33^. p38 pathway, transducing extracellular signals, coordinates the intracellular responses needed for adaption and survival. It becomes activated in response to various extra- and intracellular stress, including inflammatory cytokines and oxidative stress^50^. The current study has demonstrated that ROS activation is the main stressor that triggers p38 MAPK activation in offspring of prenatal LPS exposure following a rapid short term ISO challenge. This ROS activation mediated p38 activation ignites the ROS-p38 MAPK-NADPH oxidase-ROS positive feedback loop, leads to oxidative stress and aggravates the myocardial damage when exposed to a cardiovascular risk factor in offspring of LPS-treated mothers. There are several lines of evidence that support this concept: (i) p38 activation, in agreement with ROS activation, pre-existed in the heart tissue of offspring from LPS-treated mothers without ISO treatment. (ii) p38 over-activation occurs rapidly after 30 min of ISO treatment, and NAC can absolutely reverse this p38 activation at the end of 2 weeks of ISO treatment in offspring of LPS-treated mothers. (iii) p38 inhibitor SB202190 protected offspring of LPS-treated mothers from heart damages along with inhibited NADPH oxidase up-regulation induced by ISO treatment. This indicates that programmed ROS activation by prenatal detrimental factor exposure together with postnatal stress induced p38 activation synergistically contribute to the increased the incidence and severity of cardiovascular diseases in the adult.

Based on the current finding in our study, we would like to re-suggest a strategy based on modulating balance of ROS generation and elimination or p38 activation to prevent cardiac disease, although several clinical trials by using antioxidant^51^ or p38 inhibitor^52^,^53^ are disappointed with several speculations. Some believe that most of the individuals included in large clinical trials had significant cardiovascular disease, in which case the damaging effects of oxidative stress may be irreversible^51^. Anti-oxidant NAC or p38 inhibitor SB202190 administration together with ISO treatment during the whole duration did not show any obvious protective effect on control offspring either in our current study. In view of amplified ROS generation caused by ROS-dependent p38 activation in current model of prenatal inflammatory exposure, it indicates that we should re-think the choice standard of patients that participating in application of antioxidants or p38 inhibitor for preventing or treating cardiovascular disease. Would the indications for using antioxidants or p38 inhibitor be limited to obvious imbalance of ROS generation and elimination in the patients with cardiovascular diseases ? 

In summary, our study offered supporting evidence that maternal inflammation predisposes offspring to ISO-induced cardiac geometric, morphological and contractile abnormalities. Although it is accepted that developmental regulation of cardiovascular function is dependent on genotypes and environment, data from our study demonstrates that prenatal environmental risk factors, such as inflammation, can be an independent risk factor for adult cardiac fibrosis and function decline. Our results also suggest that positive loop between ROS and p38 MAPK activation may play a critical role in the higher susceptibility of postnatal SNS activation- induced myocardial damage in offspring of LPS-treated mothers (Fig. 8c). These findings should shed some lights towards the better management of adult stress-associated heart diseases.

## Materials and Methods

### Reagents and chemicals

LPS, ISO and NAC were obtained from Sigma (St. Louis, MO, USA); Tribromoethanol was obtained from Aldrich Chemical Co., Inc. (Milwaukee, WI, USA) and SB202190 was purchased from MedChem Express (Monmouth Junction, NJ, USA). All other chemicals were reagent grade and purchased from commercial sources and used without further purification.

### Animals and treatment

All experiments were conducted in accordance with the principles outlined in the National Institutes of Health Guide for the Care and Use of Laboratory Animals. All procedures and protocols were approved by the local animal ethics committee at Third Military Medical University. Pregnant SD rats were purchased from the Animal Centre of Third Military Medical University (Chongqing, China). All animals were provided standard laboratory rat chow and tap water. Pregnant rats were housed individually in a room at constant temperature (24 °C) and under a 12-h light–dark cycle until the end of experiments. Pups were raised with a lactating mother until 3 weeks of age, at which time they were weaned to cages containing four pups for each.

#### Study I: Prena*t*al LPS stimulation

Animals were treated as described previously^13^. Briefly, time-dated pregnant SD rats (250 g to 300 g) were randomly divided into two groups and the pregnant rats in these groups were intraperitoneally (i.p.) administered with saline (Control group) or LPS (0.79 mg/kg) (LPS group), respectively on gestation days 8, 10 and 12.

#### *Study* II*: Postnatal ISO treatment*

Pups from the aforementioned control and LPS group at the age of 20 weeks were treated with 2 weeks of ISO (5 mg/kg/day, subcutaneous injections), named as Con + ISO and LPS + ISO group, respectively. Offspring from both control and LPS group treated with saline were taken as vehicle control, identified as Con + Ve and LPS + Ve group, respectively. For antioxidant NAC treatment, offspring from both control and LPS group were subjected to NAC (2.5 g/L, estimated oral intake of 500 mg/kg/d)^54^ through drinking water simultaneously with ISO treatment, named as Con + ISO + NAC and LPS + ISO + NAC group, respectively. For inhibition of p38 activity, pups from both control and LPS group were simultaneously treated with SB202190 daily (0.4 mg/kg/day, i.p.)^55^ and ISO treatment, named as Con + ISO + p38i and LPS + ISO + p38i group, respectively. 20-week-old pups from both control and LPS group were also treated with one time of ISO (5 mg/kg) for 30 min. Pups from the same lactating mother were randomly separated into 2 to 4 groups, according to the treatments needed for each experiment.

## Echocardiography

Echocardiography was performed as previously described^16^. Rats were anesthetized with 2.5% tribromoethanol (0.25 g/kg body weight). M-mode and 2D Echocardiographic measurements were performed using a Visual Sonics Vevo^®^ 2100 Imaging System (Visual Sonics, Toronto, Canada) with a 21 MHz MicroScan transducer (model MS-550D). Cardiac function and heart dimensions were evaluated by 2D echocardiography on anesthetized rat offspring. M-mode tracings were used to measure anterior and posterior wall thicknesses at end diastole (LVPWD) and end systole (LVPWS). Left ventricular internal diameter (LVID) was measured as the largest anteroposterior diameter in diastole (LVIDD) or systole (LVIDS). LV mass and functional parameters such as percentage of left ventricular fractional shortening (LVFS%), left ventricular ejection fraction (LVEF%) and left ventricular volume were calculated using the above primary measurements and accompanying software. LVEF% was calculated as (LVEDV − LVESV)/LVEDV × 100% and LVFS% was calculated as (LVEDD − LVESD)/LVEDD × 100%.

### Masson trichrome staining

Masson trichrome staining of LV tissues was performed as previously described^15^. Briefly, LV tissues were sliced into 3–4 mm sections, which were fixed with 10% formalin (pH 7.4), embedded in paraffin, sectioned into 6-μm slices, and stained with Masson trichrome staining according to standard procedures. The slides were examined microscopically at 200X magnification. The collagen volume fraction (CVF) was determined by the area of myocardial collagen/the area of the field by using an image analysis system (Image-Pro Plus, Version 6.0; Media Cybernetics, Silver Spring, MD, USA).

### Biochemical markers of oxidative stress

To analyze the level of tissue oxidative status in left ventricular (LV), MDA level, GSH level and SOD activity in LV was quantified using commercially available kits (Nanjing Jianchen Bioengineering Institute, Nanjing, China), according to the manufacturer’s instructions.

### Real-time RT-PCR

Real-time RT-PCR was performed as previously described^15^,^56^. Total RNA was purified from isolated left ventricles with TRIzol reagent (Invitrogen) according to the manufacturer’s instructions. Concentration and purity of RNA were tested with NanoDrop ND-2000 (Thermo-Pierce, Rockford, IL, USA), OD260/280 = 1.8～2.0 is considered qualified. Total RNA (1 μg) was then reverse-transcribed into cDNA using a First Stand cDNA Synthesis Kit (DBI Bioscience, Ludwigshafen, Germany). Primer sequences for real-time RT-PCR were from reported literatures or designed by NCBI primer blast and listed in supplementary Table s1. Each real-time PCR reaction was carried out in a total volume of 10 μl with Quanti Tect SYBR Green PCR Master Mix (DBI Bioscience, Ludwigshafen, Germany) according to the following conditions: 2 min at 95 °C, 40 cycles at 95 °C for 10 s, 60 °C for 10 s, 68 °C for 15 s, 72 °C for 20 s, using ABI Prism 7700 Sequence Detector (Applied Biosystems, Agilent Technologies, CA, USA). Relative mRNA expression was calculated by normalizing the relative cycle threshold value to the control group after normalized by the internal control *β-actin*.

### Immunoblotting

Protein expression in left ventricle was determined by immunoblotting, as described previously^44^,^57^. Briefly, LV lysate was prepared by homogenizing frozen tissue in T-PER tissue protein extraction reagent (Thermo-Pierce, Rockford, IL, USA) with protease inhibitor cocktail (Sigma-Aldrich, St. Louis, MO, USA). Proteins were separated by 8–12% SDS-PAGE and transferred to a nitrocellulose membrane. After blocked by 5% non-fat dry milk for 1 hour at room temperature, the membranes were then incubated with primary antibodies overnight at 4 °C. The primary antibodies used were as followings: mouse anti-heavy chain cardiac Myosin (BA-G5) (α-MHC) (Abcam, Cambridge, MA, USA); mouse anti-skeletal slow myosin (NOQ7.5.4D) (β-MHC) (Sigma-Aldrich, St. Louis, MO, USA); rabbit anti-phospho- JNK(81E11), rabbit anti-JNK, rabbit anti-phospho-p38 MAPK (Thr180/Tyr182)(D3F9), rabbit anti-p38 MAPK, rabbit anti-phospho-ATF-2 (polyclone) (Cell signaling Technology, Beverly, MA, USA); rabbit anti-Nox2, rabbit anti-SOD1 and rabbit anti-SOD2 (Boster Wuhan, China). Anti-rabbit HRP or anti-mouse HRP (Invitrogen, Carlsbad, CA, USA) was used as a secondary antibody, followed by detection with an ECL detection kit (Merck Millipore, Billerica, MA, USA). Results were quantified by using Quantity-one software (Bio-Rad, Hercules, CA, USA).

### Measurement of O_2_^.−^ generation

To evaluate the production of tissue reactive oxygen species, fresh unfixed LV tissue samples were placed in optimum cutting temperature (O.C.T.) compound and frozen at −80 °C. Tissue segments were cut into 10 μm sections using a cryostat and placed on a glass slide. Sections were incubated in phosphate buffer saline (PBS) for 30 min at 37 °C, and then were incubated for 1 hour at 37 °C with 10 μM dihydroethidium (DHE) (Invitrogen-Molecular Probes, Eugene, OR, USA)^58^. Sections were then washed in PBS at 37 °C in a light-protected humidified chamber 3 times for 5 min, and fluorescence was detected using a 585-nm filter using Leica DM4000B microscope (Leica, Wetzlar, Germany). Fluorescence intensity analysis of DHE was measured by using an image analysis system (Image-Pro Plus, Version 6.0; Media Cybernetics, Silver Spring, MD, USA).

### Statistical analyses

A two-way ANOVA model followed by the Bonferroni post hoc test was used for multiple comparisons. Data were expressed as mean ± S.D. *P* values were adjusted for multiple comparisons using Tukey’s post hoctest. All tests were two-sided. *p* < 0.05 was considered statistically significant.

## Additional Information

**How to cite this article**: Zhang, Q. *et al*. Maternal inflammation activated ROS-p38 MAPK predisposes offspring to heart damages caused by isoproterenol via augmenting ROS generation. *Sci. Rep.* **6**, 30146; doi: 10.1038/srep30146 (2016).

## Supplementary Material

Supplementary Information
