# Supplementary material for: Maternal inflammation activated ROS-p38 MAPK predisposes offspring to heart damages caused by isoproterenol via augmenting ROS generation
Source: Sci Rep. 2016 Jul 22;6:30146. doi: 10.1038/srep30146 (PMC4957145; doi:10.1038/srep30146)
Supplement: Supplementary Information [file srep30146-s1.pdf]

Maternal inflammation activated ROS-p38 MAPK predisposes offspring to heart damages caused by isoproterenol via augmenting ROS generation

Qi Zhang[1,2*], Yafei Deng[1,2*], Wenjing Lai[1,2*], Xiao Guan[1,2], Xiongshan Sun[1,2], Qi Han[1,2], Fangjie Wang[1,2], Xiaodong Pan[1,2], Yan Ji[1,2], Hongqin Luo[1,2], Pei Huang[1,2], Yuan Tang[1,2], Liangqi Gu[3], Guorong Dan[1,2], Jianhua Yu[4], Michael Namaka[5,6], Jianxiang Zhang[1,2], Youcai Deng[1,2], Xiaohui Li[1,2]

Supplementary Materials

Supplementary Figures and Figure Legends

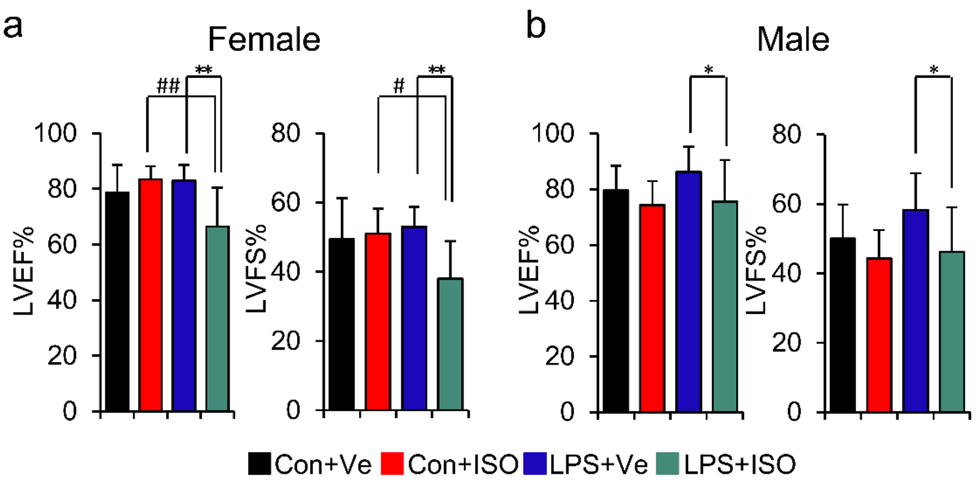

Fig. s1. Female offspring of LPS-treated mothers with isoproterenol treatment are more sensitive to systolic dysfunction than male offspring with the same treatment. Offspring were treated as described in Figure 1. At the end of ISO treatment, LVEF% and LVFS% were analyzed for both female (**a**) and male (**b**) offspring, as described in Fig. 1a. (**a**) Female offspring. n = 8 for Con+Ve; n = 6 for Con+ISO and n = 7 for LPS+Ve and LPS+ISO group. (**b**) Male offspring. n = 7 for Con+Ve; n = 5 for Con+ISO; n = 8 for LPS+Ve and n = 4 for LPS+ISO group. Error bar represents S.D. $*p < 0.05$, $**p < 0.01$, $^{\#}p < 0.05$ and $^{\#\#}p < 0.01$ denote the statistical comparison between the two marked treatment groups, respectively. One-way ANOVA analysis.

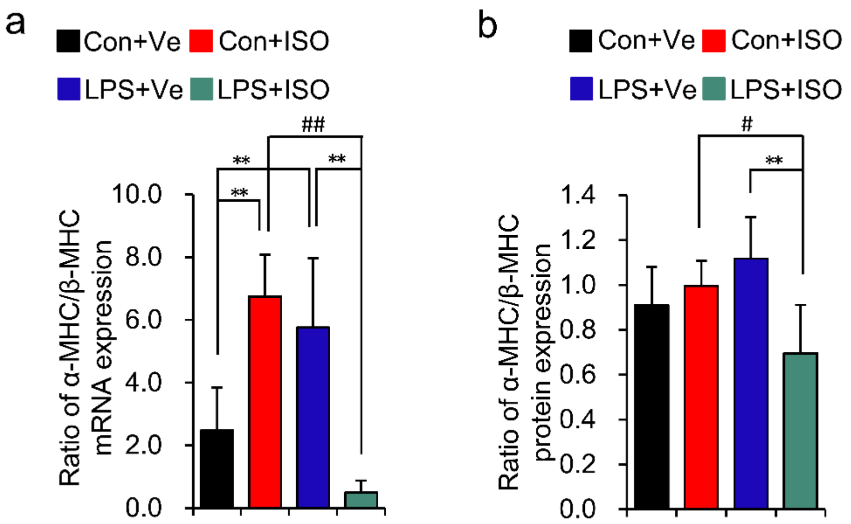

Fig. s2. Prenatal inflammation exposure leads to reduced ratio of α-MHC/β-MHC in response to ISO treatment in adult offspring. Offspring were treated as described in Figure 1. (**a, b**) The ratios of α-MHC to β-MHC (α-MHC/β-MHC) mRNA expressions (**a**) and protein expressions (**b**) in Fig. 2c and Fig. 2d, respectively, were calculated for each sample and the statistical data were shown. n = 5 offspring in each group. **$p < 0.01$, $^{\#}p < 0.05$ and $^{\#\#}p < 0.01$ denote the statistical comparison between the two marked treatment groups, respectively. Two-way ANOVA analysis.

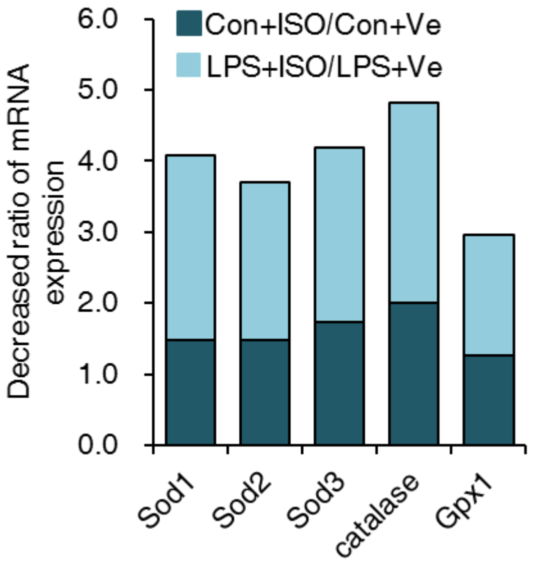

Fig. s3. Expression of antioxidant enzymes was reduced to a great extent in offspring of LPS-treated mothers after treatment with ISO. Offspring were treated as described in Fig. 1. The ratio of *Sod1*, *Sod2*, *Sod3*, *catalase,* and *Gpx1* mRNA expression, calculated by using the data acquired from with or without ISO treatment in either control or prenatal LPS-treated offspring, respectively. Dark part of the column represents the ratio of Con+ISO: Con+Ve; Light part of the column represents the ratio of LPS+ISO: LPS+Ve. n = 5 offspring in each group.

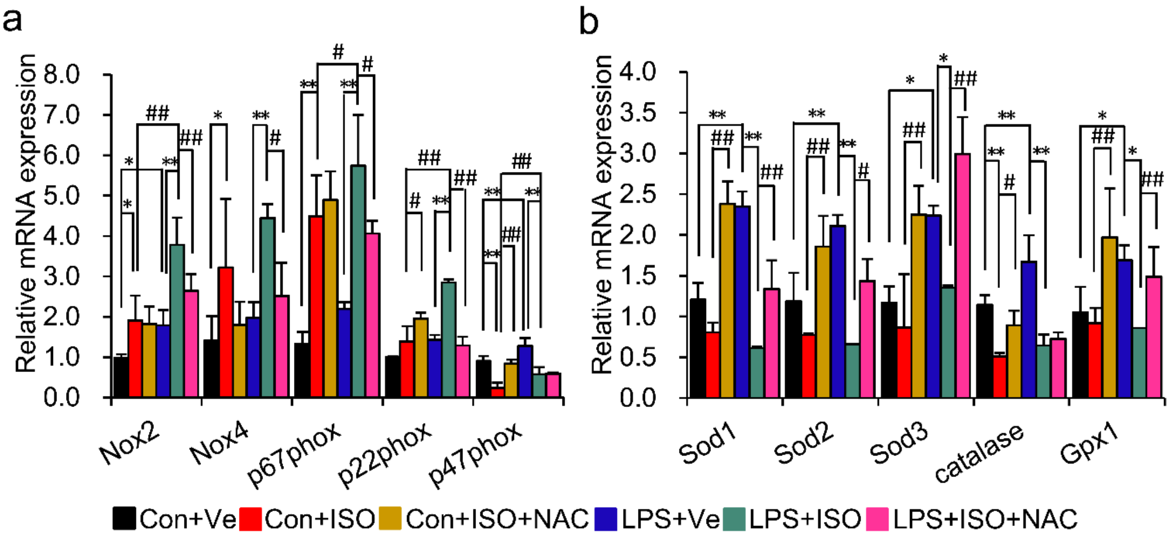

Fig. s4**. NAC reversed the mRNA expression of genes that related to ROS generation and scavenging.** Offspring were treated as described in Fig. 4. (**a, b**) The mRNA levels of NAPDH oxidase subunits (*Nox2, Nox4,* $p67^{phox}$, $p22^{phox}$ and $p47^{phox}$) (**a**) and antioxidant enzyme (*Sod1, Sod2, Sod3, catalase and Gpx1*) (**b**) in left ventricle was determined by real-time RT-PCR. *β-actin* was taken as internal control. n = 5 offspring in each group. $*p < 0.05$, $**p < 0.01$, $^{\#}p < 0.05$ and $^{\#\#}p < 0.01$ denote the statistical comparison between the two marked treatment groups, respectively. Two-way ANOVA analysis.

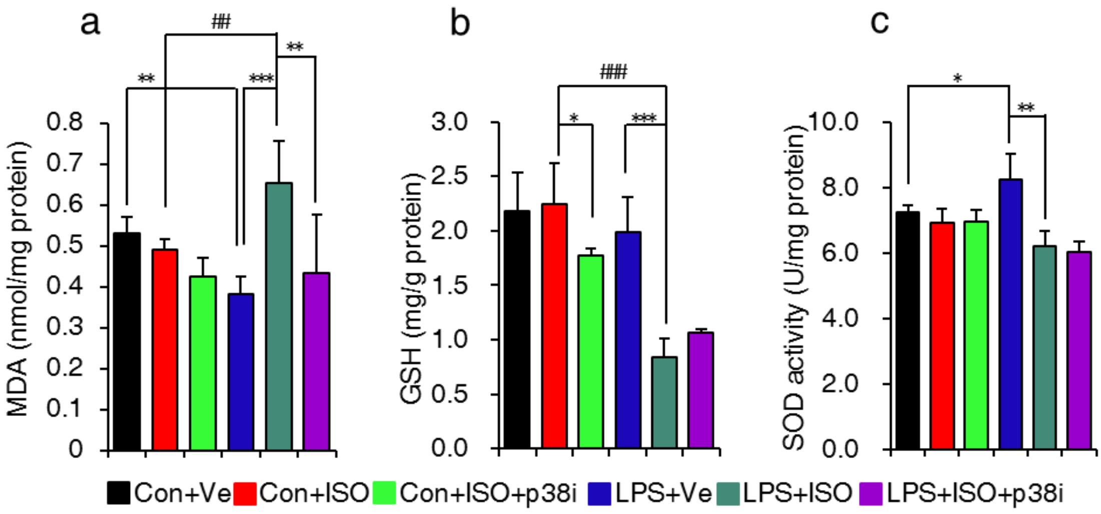

Fig. s5. SB202190 alleviated oxidative stress in offspring of LPS-treated mothers after treatment with isoproterenol. Offspring were treated as described in Fig. 7. (**a**, **b**, **c**) The level of MDA (**a**), GSH (**b**) and SOD activity (**c**) in left ventricular tissue were quantified in offspring. n = 5 offspring in each group. * $p < 0.05$, ** $p < 0.01$, *** $p < 0.001$, $^{\#\#}$ $p < 0.01$, $^{\#\#\#}$ $p < 0.001$ denote the statistical comparison between the two marked treatment groups, respectively. Two-way ANOVA analysis.

Table s1. Primers for Real-time RT-PCR

Gene symbol	Forward primer (5’-3’)	Reverse primer (5’-3’)
α-MHC[1]	GACACCAGCGCCCACCTG	ATAGCAACAGCGAGGCTCTTTCTG
β-MHC[1]	GGAGCTCACCTACCAGACAGA	CTCAGGGCTTCACAGGCATCC
ANP[1]	AGCGAGCAGACCGATGAAG	AGCCCTCAGTTTGCTTTTCA
BNP[1]	TTCCGGATCCAGGAGAGACTT	CCTAAAACAACCTCAGCCCGT
Collagen type I	TGTTCAGCTTTGTGGACCTC	CTTAGGCCATTGTGTATGC
Collagen type	CTGGTCCTGTTGGTCCATCT	ACCTTTGTCACCTCGTGGAC

III		
Mmp[2]	GATGGATACCCATTTGACGG	CCATCAGCGTTCCCATACTT
Mmp9[2]	GCAACGGAGACGGCAAACC	GACGAAGGGGAAGACGCA
Nox2	GGAGTGGTGTGTGAATGC	TTTGGTGGAGGATGTGATGA
Nox4[3]	ACAGTCCTGGCTTACCTTCG	TTCTGGGATCCTCATTCTGG
$p67^{phox}$[4]	GCTTCGGAACATGGTGTCTAAGA	AGGGTCAGGCAGTAGTTTTTCACTTG
$p22^{phox}$[5]	TGGCCTGATCCTCATCACAG	AGGCACGGACAGCAGTAAGT
$p47^{phox}$[5]	TCACCGAGATCTACGAGTTC	TCCCATGAGGCTGTTGAAGT
Sod1[4]	TGTGTCCATTGAAGATCGTGTGA	TCTTGTTTCTCGTGGACCACC
Sod2[4]	TTAACGCGCAGATCATGCA	CCTCGGTGACGTTCAGATTGT
Sod3	TTCCCAGACACCTCAATCGC	TCTGTGGAGTGCATAGGTGTG
Catalase	AAACCCGATGTCCTGACCAC	CCTTTGCCTTGGAGTATCTGG
Gpx1	TCGAACCCGATATAGAAGCCC	CACCAAGCCCAGATACCAGG
β-actin	GACGTTGACATCCGTAAAGACC	TAGGAGCCAGGGCAGTAATCT

Table s2. Echocardiography analyses of cardiac function after ISO treatment for 2 weeks in adult offspring

	Con+Ve (n=15)	Con+ISO (n=11)	LPS+Ve (n=15)	LPS+ISO (n=11)	Group *p* value	Gender *p* value
HR(bpm)	436.511±42.662	400.358±75.380	436.672±56.970	406.972±61.666	0.497	0.826
LVEF(%)	79.151±2.291	77.725±2.362	83.952±2.201^{a**}	72.569±3.855^{b***,}	0.042*	0.647
LVFS(%)	49.741±2.613	47.395±2.368	55.198±2.508^{a**}	43.699±3.277^{b***,}	0.033*	0.521
IVSD(mm)	1.724±0.300	2.623±0.401^{a***}	1.708±0.370	2.658±0.335^{b***}	0.000***	0.146
IVSS(mm)	2.816±0.374	3.747±0.330^{a***}	3.155±0.565^{a*}	3.688±0.325^{b**}	0.000***	0.029*
LVEDV(ul)	167.233±39.443	121.191±36.809^{a**}	183.513±59.865	137.533±16.171^{b**}	0.000***	0.026*
LVESV(ul)	37.536±12.144	24.716±12.193^{a*}	27.075±18.825	40.169±17.340^{b*,}	0.798	0.345
LVIDD(mm)	5.779±0.570	4.974±0.685^{a**}	5.984±0.853	5.264±0.292^{b**}	0.000***	0.064
LVIDS(mm)	3.048±0.441	2.530±0.529^{a*}	2.570±0.738^{a*}	3.036±0.569$^{b*, c\#}$	0.872	0.694
LVPWD(mm)	2.002±0.401	2.620±0.377^{a***}	1.959±0.418	2.682±0.426^{b***}	0.000***	0.264
LVPWS(mm)	3.038±0.407	3.664±0.387^{a**}	3.325±0.540	3.557±0.479	0.005**	0.043*

LVEF%: left ventricular ejection fraction; LVFS%: Fractional shortening; LVIDD: Left ventricular end diastolic internal dimension; LVIDS: Left ventricular end

systolic internal dimension; LVPWD: Left ventricular end diastolic posterior wall dimension; LVPWS: Left ventricular end systolic posterior wall dimension; LVEDV: Left ventricular end diastolic volume; LVSDV: Left ventricular end systolic volume. Error bar represents S.D. Group: group difference; Gender: gender difference. a: Con+ISO vs Con+Ve; b: LPS+ISO vs LPS+Ve; c: LPS+ISO vs Con+ISO. $^{*}p < 0.05$, $^{**}p < 0.01$, $^{***}p < 0.001$, and $^{\#}p < 0.05$. Two-way ANOVA test.

Table s3. Echocardiography analyses of cardiac function after ISO treatment for 2 weeks in female and male offspring of control and LPS-treated mothers

	Female				Male			
	Con+Ve (n=8)	Con+ISO (n=6)	LPS+Ve (n=7)	LPS+ISO (n=7)	NS+NS (n=7)	NS+ISO (n=5)	LPS+NS (n=8)	LPS+ISO (n=4)
LVEF(%)	78.719 ±10.015	83.442 ±4.78	83.031 ±5.679	66.42 ±14.012$^{b**,c\#\#}$	79.534 ±8.934	74.25 ±8.62	86.209 ±9.02	75.514 ±14.949^{b*}
LVFS(%)	49.408 ±11.85	51.024 ±7.25	52.976 ±5.725	38.006 ±10.833$^{b**,c\#}$	50.036 ±9.769	44.285 ±8.14	58.292 ±10.6	46.201 ±12.884^{b*}

LVEF%: left ventricular ejection fraction; LVFS%: Fractional shortening. Error bar represents S.D. b: LPS+ISO vs LPS+Ve; c: LPS+ISO vs Con+ISO. $^{*}p < 0.05$, $^{**}p < 0.01$, $^{\#}p < 0.05$ and $^{\#\#}p < 0.01$, One-way ANOVA test.

References

1. Nishi, H. *et al.* MicroRNA-27a regulates beta cardiac myosin heavy chain gene expression by targeting thyroid hormone receptor beta1 in neonatal rat ventricular

myocytes. *Mol Cell Biol* **31**, 744-55 (2011).

2. Mohammad, G., Mairaj Siddiquei, M., Imtiaz Nawaz, M. & Abu El-Asrar, A.M. The ERK1/2 Inhibitor U0126 Attenuates Diabetes-Induced Upregulation of MMP-9 and Biomarkers of Inflammation in the Retina. *J Diabetes Res* **2013**, 658548 (2013).
3. Zarzuelo, M.J. *et al.* SIRT1 inhibits NADPH oxidase activation and protects endothelial function in the rat aorta: implications for vascular aging. *Biochem Pharmacol* **85**, 1288-96 (2013).
4. Tina Chabrashvili, C.K., Jonathan Blau, Alex Karber, Shakil Aslam, William J. Welch, Christopher S. Wilcox. Effects of ANG II type 1 and 2 receptors on oxidative stress, renal NADPH oxidase, and SOD expression. *Am J Physiol Regul Integr Comp Physiol* **285**, 8 (2003).
5. Fan, C. *et al.* Synergy of aldosterone and high salt induces vascular smooth muscle hypertrophy through up-regulation of NOX1. *J Steroid Biochem Mol Biol* **111**, 29-36 (2008).
